# Fourth Annual Report of the Trustees of the Williamsburgh Dispensary

**Published:** 1855-05

**Authors:** 


*Fourth Annual Report of the Trustees of the Williamsburgh Dispensary of Brooklyn, Eastern District, for the year ending Feb.* 1, 1855.

"Do good unto all men as ye have opportunity." There is nothing of special interest in this report to distinguish it from a hundred others. Nothing save the old and true story of great good accomplished; of hundreds of foreigners receiving the charity without which they must have perished; of a favorable balance in the Treasurer's hands; and of who is President, or Vice, and who Secretary.

But there are "sermons in stones," and all this dry matter-of-fact detail has its lesson, and its warning if be but read aright. And we read in this report that sundry liberal gentlemen, distinguished for charity, have severally given of their wealth, $25 each to the treasury, that in all they have received from every source some $1500, of which they have by cautious economy reserved the better part as a basis for future operations; and finally, that at this trivial expense 4349 patients have received the care of three physicians, and that these three physicians have, between them, received therefor, the magnanimous sum of $100.

And now gentlemen givers of $25 each, whose names are so ostentatiously paraded as the charitable salt of the earth, what are your alms compared with those rendered by these three men? By what right do you exact of them this service, so immensely disproportioned to your gifts? Which of you has not saved in his poor-tax twice the amount he has given? And what claim to the name of charity has this plan of yours of robbing Peter to pay Paul, of drawing upon the hopes, the ambitions, or the Godlike charity of young physicians, for almost the whole support of that burthen which righteously belongs on your shoulders?

Messrs. Managers of the Williamsburgh Dispensary, you are no worse than a thousand others like you, rather better, indeed, than those almoners of legal charities 'yclept Supervisors, and Poormasters, but we have serious doubts whether these alms on which you plume yourselves so much, will not, in the light of a day that is to come, look miserably small and utterly worthless to build any claim to Heaven upon! H.

